# The role of potential oxidative biomarkers in the prognosis of intracerebral hemorrhage and the exploration antioxidants as possible preventive and treatment options

**DOI:** 10.3389/fmolb.2025.1541230

**Published:** 2025-02-04

**Authors:** Jiayong Yao, Xiaohong Dai, Xueping Yv, Lei Zheng, Jia Zheng, Binglin Kuang, Wei Teng, Weiwei Yu, Mingyue Li, Hongtao Cao, Wei Zou

**Affiliations:** ^1^ First Affiliated Hospital of Heilongjiang University of Chinese Medicine, Harbin, Heilongjiang, China; ^2^ Key Laboratory of Clinical Molecular Biology of Integrated Traditional Chinese and Western Medicine in Heilongjiang Province, Harbin, Heilongjiang, China; ^3^ Heilongjiang University of Chinese Medicine, Harbin, Heilongjiang, China

**Keywords:** oxidative stress, intracerebral hemorrhage, oxidative stress biomarkers, antioxidant therapy, stroke

## Abstract

Intracerebral hemorrhage (ICH) is a non traumatic hemorrhage that occurs in a certain part of the brain. It usually leads to brain cell damage. According to a large number of experimental research, oxidative stress is an important pathophysiological processes of cerebral hemorrhage. In this paper, we aim to determine how changes in oxidative stress biomarkers indicate the damage degree of cerebral hemorrhage, and to explore and summarize potential treatments or interventions. We found that patients with cerebral hemorrhage are characterized by increased levels of oxidative stress markers, such as total malondialdehyde (MDA), F2 isoprostaglandin, hydroxynonenal, myeloperoxidase and protein hydroxyl. Therefore, the changes of oxidative stress caused by ICH on these markers can be used to evaluate and diagnose ICH, predict its prognosis, and guide preventive treatment to turn to antioxidant based treatment as a new treatment alternative.

## 1 Introduction

Intracerebral hemorrhage (ICH) is a serious neurological disease with high morbidity and mortality worldwide (GBD 2019 Stroke Collaborators, 2021). The global economic burden of stroke is estimated to exceed 721 billion US dollars, representing approximately 0.66% of the global GDP (Feigin et al., 2022). On a global scale, the absolute incidence of stroke has risen by 37%, with hemorrhagic stroke accounting for 47% of all cases (GBD 2016 Neurology Collaborators, 2019). As a destructive form of stroke, ICH has a higher disability rate (Gong et al., 2021).

The risk factors of ICH are usually divided into unalterable risks (previous history of ICH, age, gender, race, cerebral amyloid angiopathy, chronic kidney disease, congenital coagulation dysfunction, etc.) and changeable risk factors (hypertension, coagulation dysfunction, current smoking, excessive drinking, diabetes, sympathetic nerve/illegal drugs, etc.) (Larsson et al., 2024). According to statistics, only 6 out of every 10 ICH patients can survive 1 month after the onset (Stokum et al., 2021), and two-thirds of the survivors are moderately or severely disabled (Chen et al., 2021a). Survivors also have motor, sensory or cognitive impairment, which ultimately affects cognitive, work and social abilities (Shi et al., 2021).

Although many preclinical and clinical trials have been completed in the past decades, and these trials have clarified the potential causes and mechanisms of cerebral hemorrhage injury, including the correlation between edema, apoptosis and oxidative stress (Li Y. et al., 2021; Li et al., 2020; Bobinger et al., 2018; Helmuth et al., 2023). However, no treatment has been proved to significantly improve the mortality and neurological prognosis after ICH. ICH surgical treatment focuses on risk factor management and prevention of deterioration after initial bleeding (Hanley, 2009; Mendelow, 2015). Many trials investigated the best drug and surgical management of ICH, but did not significantly improve the survival rate and functional outcome (Magid-Bernstein et al., 2022a; Chen P. et al., 2022; Kellner et al., 2021). Therefore, the prospect of ICH treatment is more based on primary prevention, ultra early hemostasis treatment and injury protection by pathobiology and prognostic biomarkers.

Oxidative stress (OS) is a serious imbalance between the generation of large amount of oxidative active substances and the antioxidant system in organisms. Common oxidative active substances include reactive oxygen species (ROS) and reactive nitrogen species (RNS) (Yoshikawa and You, 2024). Following ICH, the metabolism of hemoglobin released from the hematoma results in substantial iron accumulation, disrupting cellular iron homeostasis. This disruption further impairs mitochondrial function and promotes the generation of ROS (Yan et al., 2023a). The pathological injury mechanism after ICH mainly includes primary injury and secondary injury. The term refers to the initial physical damage to brain structures caused by rupture of small arteries and hemorrhage, along with the rise in intracranial pressure resulting from the mass effect of the hematoma. Secondary injury refers to the damage of toxic substances released by hematoma to the tissues around hematoma, which will further aggravate the tissue injury and neurological function defect after ICH (Hilkens et al., 2024). Oxidative stress induced neuronal damage is a major factor in the secondary injury process after ICH (Magid-Bernstein et al., 2022b). After ICH, hematoma can lead to rapid and continuous increase of intracranial ROS, leading to neuronal death, and finally secondary neurological dysfunction (Zhang et al., 2022). ROS can directly attack lipids, proteins, nucleic acids and other macromolecules to cause corresponding damage, and can also aggravate tissue damage by activating inflammatory response (Jomova et al., 2023). Due to the high demand for oxygen by neuronal cells to produce ATP via the mitochondrial respiratory chain in order to sustain normal cellular function, this process also generates substantial precursor molecules for the production of ROS (Feng et al., 2024a). Furthermore, brain tissue is particularly susceptible to oxidative damage due to its high content of polyunsaturated fatty acids, which are prone to ROS-induced oxidation. This makes the brain less equipped with robust antioxidant defense mechanisms (Yan et al., 2023b). The brain’s high oxidative metabolic rate, coupled with its low antioxidant capacity, makes it highly susceptible to ROS-induced damage. This leads to structural and functional abnormalities in mitochondria, thereby initiating the mitochondrial apoptosis pathway (Xie et al., 2020a; Zhao et al., 2024; Wang M. et al., 2022).

The main source of ROS is the mitochondrial electron transport chain and enzymatic reactions catalyzed by various enzymes (Cheung and Vousden, 2022). The superoxide anion produced by mitochondria is a by-product of the electron leakage of mitochondrial respiratory chain complex. Both superoxide anion and its product hydrogen peroxide are considered to be mitochondrial ROS (mtROS) (Okoye et al., 2023). During the pathogenesis of ICH, impaired energy supply leads to a partial loss of ion channel function in maintaining the electrochemical gradient across the cell membrane. This results in an influx of calcium ions, a reduction in the selective permeability of the mitochondrial permeability transition pore, and the influx of ions, ROS, and other small molecules into the cell. Consequently, mitochondrial dysfunction occurs, accompanied by excessive accumulation of oxidative free radicals, contributing to oxidative damage (Di et al., 2023).

Beyond mitochondria, NADPH oxidase (NOX) and myeloperoxidase (MPO) are also critical sources of superoxide anions, which serve as key reactive free radicals following ICH. NOX is a specialized enzyme that regulates the generation of ROS. ROS generated by NOX function as secondary messengers, participating in the regulation of cell differentiation, proliferation, and apoptosis. The NOX2 and NOX4 isoforms are particularly important in modulating both the physiological and pathological processes in brain tissue (Mamelak, 2024). In an experimental model of ICH in rats, upregulation of NOX4 expression was observed, which may contribute to the disruption of oxidative stress balance in brain cells (Xie et al., 2020b). Other studies have confirmed that the increase of NOX2 aggravates the oxidative damage after ICH (Wang et al., 2018).

As another important source of ROS in ICH, MPO enhances the reactivity of H_2_O_2_ by producing hypochlorite, free radicals and RNS (Lin et al., 2024). When MPO breaks out in neutrophil respiration, heme is required as a cofactor and hypochlorite is produced from the reaction of H_2_O_2_ and chloride (Marcinkiewicz and Walczewska, 2020). MPO tends to aggravate brain injury (Manoharan et al., 2024). Animal experiments found that the expression of MPO protein in rat brain tissue increased after ICH, and inhibition of MPO can alleviate ICH induced brain injury (Zuo et al., 2022a).

**FIGURE 1:**
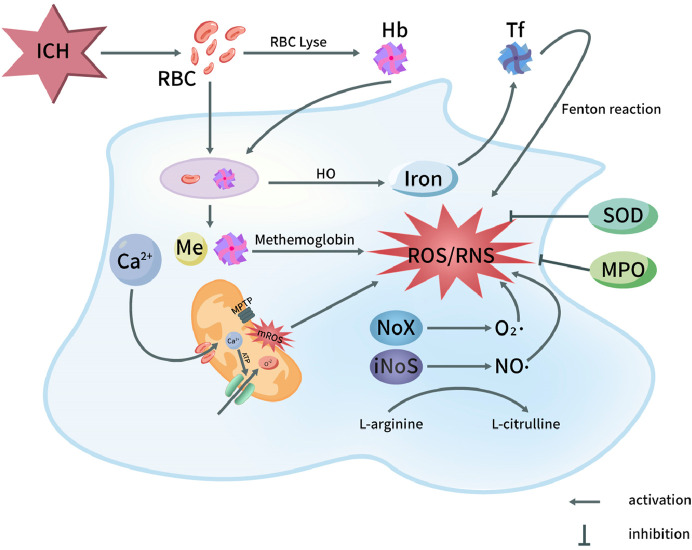
Oxidative stress pathways in pathophysiology of ICH. After ICH, the destruction of red blood cells in the hematoma releases hemoglobin and heme. Both red blood cells and degraded heme components are phagocytosed, leading to the oxidation of hemoglobin into methemoglobin, which subsequently generates reactive oxygen species (ROS). Iron is transported into the nervous system, ultimately triggering the Fenton reaction and producing ROS. On the other hand, the opening of the mitochondrial permeability transition pore leads to the release of ROS, with Ca^2+^ influx playing a role in this process. Additionally, the activation of NOX and NOS in microglial cells results in the production of superoxide (O_2_·) by NOX and nitric oxide (NO·) by NOS through the conversion of L-arginine to L-citrulline. This activation contributes to the generation of ROS and reactive nitrogen species (RNS). Furthermore, myeloperoxidase (MPO) and superoxide dismutase (SOD) exert inhibitory effects on the production of ROS/RNS. Hb, Hemoglobin; HO, Heme oxygenase; NOS, Nitric oxide synthase; ICH, Intracerebral hemorrhage; MPTP, Mitochondrial permeability transition pore; RBC, Red blood cell; Tf, Transferrin; NO, Nitric oxide; O_2_·, Superoxide anion radical; ROS, Reactive oxygen species; NOX, NADPH oxidase; MPO, Myeloperoxidase; mROS, Mitochondrial reactive oxygen species; RNS, Reactive nitrogen species; SOD, Superoxide dismutase.

Other major enzyme pathways that catalyze ROS release are endoplasmic reticulum, hemoglobin, ferrous and ferritin. The metabolism of hemoglobin released from hematoma after ICH will cause serious iron ion overload. Excessive free iron release generates superoxide anion and highly active hydroxyl radical through Fenton and Haber Weiss reaction, leading to imbalance of brain iron homeostasis and promoting ROS (Zhu et al., 2021). In addition, a large number of free radicals are produced under the induction of products after the decomposition of blood cells, such as thrombin and heme. These reactions produce ROS, such as O_2_^−^ and HO^−^ (Xu et al., 2023). ICH can cause endoplasmic reticulum stress (ERS), and decreased expression of c/ebp homologous protein (CHOP) will lead to the secondary increase of ROS level (Chen et al., 2021b). Other factors, in the inflammatory reaction process after ICH, some inflammatory cells are stimulated and activated, resulting in the release of a large number of ROS, no and other substances, and the consumption of a large number of superoxide dismutase (SOD) and lipid peroxide (Cheng et al., 2023) (Figure 1).

Recent studies indicate that a single episode of ICH can result in varying degrees of disability, including motor impairments, language deficits, and, in some cases death (Sembill et al., 2021). ICH is still a disease with high morbidity and mortality (Gómez-González et al., 2021). Therefore, understanding the underlying mechanisms of oxidative damage following ICH is crucial for reducing associated mortality. However, it is important to acknowledge that changes in the levels of redox biomarkers are observed in a variety of diseases, making it challenging to specifically associate any single biomarker with ICH. Furthermore, alterations in the body’s redox balance can be assessed both qualitatively and quantitatively through biomarkers, which provide valuable information for disease prognosis. Consequently, redox biomarkers are currently considered vital tools for monitoring the progression of ICH and its prognosis (Shao et al., 2019).

At the same time, the specific correlation between various oxidative biomarkers and ICH has been successfully confirmed (Diao et al., 2024). However, a major challenge lies in the direct detection of ROS due to their high reactivity and extremely short half-life. In other neurological disorders, OS levels have been inferred from the measurement of oxidation products in DNA, proteins, and lipids as indirect indicators (Wang L. et al., 2024). Recent study showed anti OS is an important measure to improve the neurological function of ICH and affect the prognosis (Tang et al., 2023a; Shirzad et al., 2023; Liao et al., 2024). The detection of oxidative biomarkers is not only important for the diagnosis and prognosis of ICH, but also essential for the treatment of ICH. Therefore, to explore effective laboratory indicators for evaluating the severity of oxidative stress after ICH can provide an effective means for early diagnosis or efficacy evaluation of secondary brain injury.

Biomarkers are measurable indicators used to objectively and accurately distinguish between normal biological status and pathological status, and to respond to specific treatment interventions. This review examines the potential role of oxidative stress biomarkers in the diagnosis and prognosis of ICH, as well as their utility in predicting disease outcomes. The aim is to improve diagnostic accuracy, optimize treatment strategies, enhance health outcomes.

## 2 Lipid peroxidation

DNA, Oxidation products of proteins and lipids were recorded as indirect readings of OS levels. Lipid peroxides are key products resulting from brain injury. They primarily originate from the secondary peroxidation of unsaturated fatty acids in membrane phospholipids (Zhou SY. et al., 2020). As one of the harmful products of oxidative damage, like ·OH and other free radicals, it can extract hydrogen ions on fatty acids to further generate new lipid peroxides, so once the reaction is started, it will be cascaded and amplified to produce chain reaction (Lee et al., 2023). Oxidative damage to lipids was evaluated using both quantitative and qualitative assessments of several biomarkers, including MDA, 4-hydroxy-2-nonenal (HNE), oxidized LDL, and F2 isoprostaglandin, measured in blood, cerebrospinal fluid, or other relevant bodily fluids. At present, lipid peroxides in peripheral blood are widely regarded as a reliable marker for assessing oxidative stress.

### 2.1 Malondialdehyde

MDA is the main end product of lipid peroxide and has cytotoxicity. It can react with proteins and enzymes to destroy the structure of brain tissue and dysfunction (Tsikas, 2023). Its formation arises from the destruction of brain cells and the oxidation of polyunsaturated fatty acids into peroxides, followed by their degradation (Wei et al., 2024). Due to its involvement in lipid peroxidation, MDA is the most commonly used biomarker for assessing oxidative damage and is widely recognized as a key indicator of oxidative stress (Thakkar et al., 2024).

MDA is easy to react with primary ammonia of biological macromolecules, so it exists in free state and bound state *in vivo* (Moldogazieva et al., 2023). The conventional method for the determination of free MDA or bound MDA is thiobarbituric acid (TBA) colorimetric analysis; Although TBA can also react with other unsaturated aldehydes, more than 85% of the products produced by TBA reaction (TBARS) come from the addition polymerization of MDA and TBA molecules (Jiménez-Jiménez et al., 2024). Other methods for determining MDA include high performance liquid chromatography and immunological methods (Mas-Bargues et al., 2021) (Table 1).

**TABLE 1:** Approaches for detecting biomarkers of oxidative stress.

Oxidative stress biomarkers	Detection strategy examples	References
Malondialdehyde	SERS TBARS Test Fluorescence UV-Vis GC-MS Electrochemistry	Mas-Bargues et al. (2021), Toto et al. (2022)
4-Hydroxy-2-Nonenal	MALDI-TOF-MS HPLC DNPH Derivatization 2-AP LC-MSMS Western blot 32P-PostlabelingGC-MS Sandwich ELISA FT-ICR MS	Fuloria et al. (2020), Žarković et al. (2024), Zhou et al. (2020b)
F2-isoprostanes	SPE-HPLC-MS/MS ELISA GC-MS HPLC-MS/MS GC-NICI-MS	Fuloria et al. (2020), Zhou et al. (2022), Zhou et al. (2023)
8-Iso-Prostaglandin F2α	HPLC-ED	Steffensen et al. (2020)
LOX-1	Western blot ELISA Flow cytometry analysis Fluorescence	Deng et al. (2024), Sánchez-León et al. (2024), Ashok et al. (2024), Bing et al. (2024)
8-oxo-7	Fluorescence HPLC-ED ELISA	Dong et al. (2024a), Nian et al. (2024), Fujikawa et al. (2024)
Protein carbonyls	Levine spectrophotometric method In-gel fluorophoric tagging ELISA Western blot HPLC	Ladouce et al. (2023), Zhang et al. (2025), Nocera et al. (2024)
RAGE	Western blot ELISA	Han et al. (2024), Vega-Cárdenas et al. (2023), Kanikowska et al. (2024)
Homocysteine	Immunonephelometric method HPLC-ED EIA LC-MS-MS Immunoassay HPLC with fluorometric detection Fluorescence polarization	Alam et al. (2019)
Gluthatione	DTNB/GR enzyme recycling method HPLC	Added et al. (2023), Carrão Dantas et al. (2022)
GFAP	Spectroscopy Fluorescence polarization	Probert et al. (2022), Pereira et al. (2021)
HMGB1	Western blot LC-MS-MS ELISA	Held et al. (2024)
IMA	Western blot LC-MS-MS	Li et al. (2022), Karakılıç et al. (2023)
S100B	ELISA HPLC-MS/MS Fluorescence	Bjursten et al. (2024)
AOPP	ELISA Fluorescence HPLC-MS/MS	Li et al. (2024), Wybranowski et al. (2023)
Myeloperoxidase	BLI CRET ADHP MPO-Gd MR imaging	Gramstad et al. (2023), Majid et al. (2023)

SPE, polymeric weak anion-exchange solid-phase extraction; TBARS, thiobarbituric Acid Reactive Substances; GC-MS, gas chromatography-mass spectrometry; UHPLC–MS/MS, isotope-dilution ultrahigh performance liquid chromatography electrospray ionization–tandem mass spectrometry; NICI-MS, gas chromatography-negative-ion chemical ionization mass spectrometry; HPLC, high performance liquid chromatography; HPLC-ED, high performance liquid chromatography with electrochemical detection; EIA, enzyme-linked immunoassay; ADHP, 10-acetyl-3, 7-dihydroxyphenoxazine; 2-AP, fluorescent probe 2-aminopyridine; CRET, chemiluminescence resonance energy transfer; DNPH, 2,4-dinitrophenylhydrazine; BLI, bioluminescence imaging; MPO-Gd, bis-5-hydroxytryptamide-diethylenetriaminepentaacetate-gadolinium; measurement detection spectroscopy.

Regarding the relationship between MDA and ICH, recent studies indicate that serum levels of MDA may have a positive correlation with changes in neurological function associated with ICH (Akyol et al., 2024).

The formation of MDA and TBARS is not solely attributed to oxidative stress, but also results from the degradation of endogenous epoxides. Therefore, MDA assessment extends beyond blood samples and is often used in conjunction with routine measurements of serum total antioxidant status (TAS) as a more comprehensive indicator of oxidative balance. Masomi et al. Suggested that markers with increased OS could also be detected in cerebrospinal fluid (CSF) and plasma, and higher MDA levels were detected in cerebrospinal fluid of a large number of ICH patients, leading to adverse results after 6 months (Masomi-Bornwasser et al., 2021a).

### 2.2 4-Hydroxy-2-nonenal (4-HNE)

Protein oxidation induced by ROS can result in the formation of carbonyl derivatives and nitrated tyrosine residues, with 4-HNE being a key byproduct. Among lipid aldehydes, 4-HNE is considered one of the most biologically active (Jaganjac et al., 2022). 4-HNE is an advanced lipid peroxidation end product (Lamb et al., 2024). 4-HNE is directly involved in cytotoxic processes and is strongly associated with oxidative damage (Yamashima et al., 2024; Lee et al., 2024). Like MDA, it can react with various cell components and can also be used as an indicator of lipid peroxide *in vivo*, and is considered to be one of the most powerful active aldehydes (Zhang Y. et al., 2024). Animal studies indicated that the accumulation of 4-HNE progressively increased over the 3–18 days following ICH (Wang et al., 2022b). It is reported that Chen et al. Used rats as experimental models to inhibit 4-hne-related oxidative stress through edaravone, thus providing neuroprotective effect after ICH (Chen Q. et al., 2022). A clinical study, in comparison with animal model findings, demonstrated that levels of 4-HNE in blood samples were significantly elevated in all ICH patients compared to non-ICH subjects, providing valuable insights into the oxidative stress-related consequences following ICH (Jarocka-Karpowicz et al., 2020a).

### 2.3 F2-isoprostanes and 8-iso-Prostaglandin F2 alpha

F2 isoprostanes are a series of prostaglandin F2 like compounds produced by lipid peroxidation reaction of arachidonic acid on cell membrane by ROS (Mottola et al., 2024). From the varying impacts of reactive oxygen species on arachidonic acid locations, a total of 64 isomers of F2 isoprostane may be produced (Cioffi et al., 2021). 15-F2t-isoprostane is the most studied and most representative isomer of F2 isoprostane (Simantiris et al., 2023). In recent years, F2 isoprostaglandin has emerged as a reliable biomarker for oxidative stress and lipid peroxidation *in vivo*, attributed to the following characteristics:① F2 isoprostaglandin is a specific byproduct of lipid peroxidation that does not require cyclooxygenase for its formation, and it possesses relatively stable chemical properties and content (Katsioupa et al., 2023); ② In some animal models of oxidative damage, its level increased significantly; ③ Its level is not affected by eating lipids (Trares et al., 2022). Currently, F2 isoprostaglandin is recognized as among the most dependable markers for assessing oxidative stress *in vivo* (de Mello Barros Pimentel et al., 2023). Recent studies have shown that the level of F2 isoprostaglandin has a certain correlation with free radicals and oxidative damage in many human diseases (including cardiovascular diseases, lung diseases, nervous system diseases, kidney and liver diseases, etc.) (Menzel et al., 2021). In their investigation of F2 isoprostaglandins for prognostic purposes in ICH, Gomes et al. reported that specific markers of *in vivo* lipid peroxidation, namely, F2 isoprostaglandins (F2 IsoPs) and isoprostaglandins (isof), were elevated and influenced prognosis following SAH (Gomes et al., 2022). Another study also showed that F2 isoprostaglandin can be used as a non-invasive prognostic biochemical marker for ICH patients (Wiśniewski et al., 2017). Nevertheless, while F2 isoprostaglandins are generally reliable biomarkers for forecasting stroke outcomes, they do possess certain limitations. Because the formation of F2 isoprostaglandin is disturbed under high oxygen tension, F2 isoprostaglandin is not sensitive enough as an indicator of lipid peroxidation under high oxygen tension (Thatcher and Peters-Golden, 2022).

#### 2.3.1 8-Iso-prostaglandin F2α

8-Isoprostaglandin F2α is widely present in the body, and its formation occurs independently of cyclooxygenase catalysis, which is why it is named for its structural similarity to prostaglandins. As the most representative isomer of isoprostaglandin, both side chains are CIS structure, which is the most widely studied isoprostaglandin (Boldeanu et al., 2023). Due to the stable structure *in vivo*, the content of isoprostaglandin is not affected by the lipid in food, with good sensitivity and specificity, and is closely related to oxidative stress injury, it is currently considered to be an ideal index for evaluating the oxidative stress state of the body (Zhang M. et al., 2021). Recent studies indicate that the levels of 8-Isoprostaglandin F2α increase in response to oxidative stress following ICH (Yang et al., 2021). In their clinical investigation of the prognosis and outcomes of ICH, Du et al. observed a increase in plasma levels of 8-Isoprostaglandin F2α in ICH patients. Furthermore, these elevated levels were positively correlated with hematoma volume and associated with unfavorable clinical outcomes in ICH (Du et al., 2014).

### 2.4 Lectin-like oxidation of the LDL receptor-1 (LOX-1)

LOX-1 is widely expressed in endothelial cells, where it can activate multiple cell death pathways, elevate reactive oxygen species, and contribute to endothelial dysfunction (Akhmedov et al., 2021). LOX-1 is expressed not only on the surface of cells but also exists as soluble molecules within the bloodstream (Pyrpyris et al., 2024). There are case reports that the expression of LOX-1 and matrix metalloproteinase can be significantly increased in the intima of ruptured and unruptured middle cerebral artery, and the expression level of s LOX-1 in patients with acute aortic dissection is extremely increased, which is speculated to be related to the rapid and severe damage of vascular endothelium (Kobayashi et al., 2013). ICH, LOX-1 binds to red blood cells, leading to the upregulation of both LOX-1 and sLOX-1expressions. Inoue et al. reported that serum levels of LOX-1 were elevated in ICH patients, based on analysis of blood samples, suggesting that LOX-1 could serve as a potential biomarker for ICH (Inoue et al., 2019). Similarly, a clinical study conducted by Yokota et al. found that, compared to healthy individuals, the levels of soluble LOX-1 increased in patients following the onset of ICH. This finding supports the use of elevated sLOX-1 levels as a potential biomarker for ICH (Yokota et al., 2016).

## 3 DNA oxidation

In biological macromolecules, proteins, lipids, and RNA typically undergo degradation and recycling following oxidative damage, whereas DNA requires repair to preserve genomic integrity (Draxler et al., 2023). Oxidative damage to DNA includes various forms such as base oxidation and deoxyribose oxidation, with base oxidation being the predominant type of damage (Andrés et al., 2023). Among the four nucleobases, guanine exhibits the lowest redox potential, making it particularly susceptible to oxidative damage (Gupta and Imlay, 2023). As an oxidative adduct, 7,8-dihydro-8-oxoguanine (8-oxoG) is stable within the body. Once formed, it is not further metabolized and is not influenced by dietary intake or other external factors. It can be excised and removed by specific DNA repair enzymes and subsequently excreted in urine via the kidneys. (Dong et al., 2024b). Therefore, 8-oxo-7 may be a sensitive biomarker of brain DNA damage driven by oxidative stress (Shkirkova et al., 2024).

In previous preclinical and clinical studies on central and peripheral nerve disorders and mental disorders, it was found that its content increased with the increase of brain injury (Schiavone et al., 2017). The study found that after ICH, the expression of 8-oxo-7 in brain tissue increased significantly (Liu et al., 2020). In a recent observational prospective study by Lorente et al., the average urinary levels of 8-oxo-7 in 100 stroke patients on day 7 were higher than those in the control group. Furthermore, the authors found a positive correlation between 8-oxo-7 levels and mortality, based on comparisons of serum samples from patients with ICH and their 30-day mortality outcomes (Lorente et al., 2020).

## 4 Oxidative protein modifications

The oxidation of amino acids in protein composition usually has reversible and irreversible effects on the function and structure of the affected proteins, which makes protein oxidation products a potential biomarker of ICH oxidative damage. This section will discuss the relationship between common protein oxidation products and ICH.

### 4.1 Protein carbonyls

Protein carbonylation is one of the oxidative damage of proteins. It is an irreversible chemical modification in oxidative stress, which means that the side chain of amino acid residues is attacked by oxygen free radicals and finally transformed into carbonyl products (Nègre-Salvayre and Salvayre, 2024). There are two major pathways for the formation of carbonyl proteins: (1) ROS directly oxidizes the side chain amino acids of proteins to form carbonyl proteins (i.e., free radical oxidation); (2) proteins generate carbonyl proteins (i.e., glycosylation); after lipid oxidation and nonenzymatic glycosylation (Wang et al., 2023). Utilizing an *in vivo* metal ion catalytic oxidation system, ROS can directly oxidize protein side chain residues, including lysine, arginine, proline, and threonine, leading to the formation of carbonyl groups (Gulyak et al., 2023). Therefore, protein carbonylation as a necessary marker of OS is of great significance because of its sensitivity and specificity (Chen et al., 2023).

There are relatively few studies on protein carbonyl as an oxidative marker, and there is a lack of sufficient scientific data. One common reason is that although lc-ms/ms is an established method for detecting and quantifying oxidative markers (including carbonylated proteins), its huge cost and the demand for a large amount of expertise limit its wide adoption (Martínez-Orgado et al., 2023a). Martínez-Orgado et al. proposed a potential correlation between plasma protein carbonyl levels and brain injury, suggesting that these carbonyl compounds could serve as biomarkers for the extent of brain damage induced by oxidative stress (Martínez-Orgado et al., 2023b). A recent clinical study on the risk factors of aneurysm size, multiple, previous SAH history and oxidative stress showed that when SAH and aneurysm rupture occurred, myeloperoxidase activity, malondialdehyde and carbonyl levels increased significantly (Šćepanović et al., 2018). It is suggested that carbonyl determination may be a potential marker of oxidative stress after ICH.

### 4.2 Receptors for advanced glycation end products (RAGE)

In recent years, RAGE and its soluble form (sRAGE) have increasingly been recognized as biomarkers for ICH (Balança et al., 2021). The RAGE interacts with various ligands, including HMGB-1, advanced glycation end-products (AGEs), and S100 proteins. The expression of RAGE is associated with oxidative stress induction, while sRAGE exerts a protective effect by competing with RAGE for ligand binding (Reddy, 2023). Lei et al. Carried out an interesting study, revealing the relationship between the soluble RAGE level and the severity of ICH, as well as the significant increase in adverse functional outcomes (Lei et al., 2020). Another study found that the level of sRAGE in SAH patients increased early and then changed dynamically, which may be a potential biomarker of poor prognosis, providing more accurate prognostic information for sRAGE quantification after ICH (Chu et al., 2023).

### 4.3 Homocysteine

Homocysteine is produced by adenosine transfer, demethylation and hydrolysis under the catalysis of enzyme. It is metabolized by re methylation or trans sulfur under the action of folic acid and vitamin B group (Grande et al., 2023). Homocysteine exists in the form of the plasma in four different forms: about 1% in plasma in free disulfide homocysteine, 70%–80% is keeping disulfide and plasma protein binding (mainly albumin), 20%–30% is disulfide bond form dimer, and a few in the form of other thiol-containing group in plasma. The concentration of plasma homocysteine that we usually measured refers to the total concentration of (Zhilyaeva et al., 2023). Homocysteine in plasma predominantly exists in its oxidized state, with only a minor fraction present in its reduced form. The auto-oxidation of homocysteine is a well-established pathway for ROS generation (An et al., 2024). Homocysteine possesses a reactive thiol group, which is readily subject to auto-oxidation, leading to the generation of various reactive oxygen species (Gawel et al., 2024). The sulfhydryl group of homocysteine can also undergo one electron oxidation reaction to generate sulfur free radical or disulfide anion free radical with another compound containing sulfhydryl group, and these two free radicals are more likely to cause protein oxidation (Zarembska et al., 2023). As a cell injury factor, the main mechanism of homocysteine is to induce the occurrence of oxidative stress (Guo et al., 2024). A clinical study involving 551 patients with ICH found that elevated homocysteine levels were present in 284 patients (51.5%). Significant differences in the percentage of males, smoking and drinking behaviors, and triglyceride levels were observed among the different homocysteine level groups. In ICH patients, higher homocysteine levels were associated with reduced survival rates and worse prognoses (Wang D. et al., 2020). A recent multicenter, hospital-based study conducted nationwide in China by the China Stroke Center Alliance (CSCA) assessed 705 patients with ICH. The results revealed a significant association between elevated homocysteine levels and both the severity of ICH at admission and unfavorable functional outcomes at discharge. These findings suggest that homocysteine may be a useful biomarker for predicting the severity of ICH and functional prognosis at discharge (Wang et al., 2022c). Li et al. Carried out a 6-month clinical experiment involving 84 subjects with peripheral blood NLRP3 mRNA and Hcy as observation indicators, suggesting that serum Hcy, blood loss and ventricular system permeability are independent risk factors for poor prognosis in ICH patients (Li Q. et al., 2021).

### 4.4 Glutathione

Glutathione is condensed from glutamic acid, cysteine and glycine (Lapenna, 2023). Glutathione plays a crucial role as an intracellular regulatory metabolite by activating various enzymes, thereby influencing cellular metabolic processes (Ferreira et al., 2023). Glutathione participates in *in vivo* oxidation-reduction processes and can effectively interact with peroxides and free radicals, protecting sulfhydryl groups in antioxidants. It safeguards sulfhydryl-containing proteins and enzymes in cell membranes from damage and mitigates the harmful effects of free radicals on vital organs (Ichihara et al., 2023). Under the influence of oxidants, glutathione (GSH) and its oxidized form (GSSG) can interconvert through enzymatic action, establishing a dynamic equilibrium that contributes to an effective antioxidant system (Fernández-Lázaro et al., 2024). As iron death has become a recent research hotspot, the oxidative damage mechanism of GSH has been repeatedly verified in animal experiments (Nie et al., 2024). In the human study, jarocka et al. (Jarocka-Karpowicz et al., 2020b) reported that the plasma GSH level of 30 subjects increased within 6–8 days of SAH compared with the matched control group. Similarly, Akyol et al. conducted a clinical study involving 200 subjects and observed that, compared to the control group, GSH levels increased following ICH. Moreover, the GSH levels in ICH patients who underwent decompression surgery were found to be comparable to those of patients who did not receive surgery (Akyol et al., 2024). These results underscore the feasibility of GSH as a biomarker for oxidative damage in ICH.

### 4.5 Glial fibrillary acidic protein (GFAP)

GFAP is a cytoskeletal protein existing in astrocytes, which has two forms of soluble protein and intermediate filament protein. It is used to maintain the morphology and function of astrocytes, but also to protect and supply neurons. It is very sensitive to oxidative stress damage, and has been used as a clinical oxidative stress related molecule for monitoring (Passos et al., 2022). Bhatia et al. reported that collecting blood samples within 24 h of ICH episode revealed significantly elevated serum content of GFAP in ICH patients (Bhatia et al., 2020). Gyldenholm et al. also reported on a study involving 156 participants, in which GFAP levels were measured using ultra-sensitive single molecule array and ELISA, while clinical data, including mortality and functional outcomes, were recorded. The study found that serum GFAP levels were elevated in patients with ICH compared to healthy controls, and that the increase in GFAP levels was predictive of both mortality and poor prognosis in ICH patients (Gyldenholm et al., 2022).

### 4.6 High mobility group box1 (HMGB1)

HMGB1 is mainly involved in cell differentiation, stabilizing chromatin structure, regulating gene transcription and translation, and steroid hormone regulation and other life activities (Chen R. et al., 2024; Zhu et al., 2024). Nuclear HMGB1 is nonspecifically bound to DNA with low affinity, and is involved in cell differentiation, DNA repair, DNA recombination, steroid hormone regulation and other life activities in the nucleus (Tang D. et al., 2023). Rage mentioned above is a receptor in the traditional signal transduction pathway of HMGB1, which belongs to the immunoglobulin superfamily and is expressed on a variety of cell surfaces (Lu Z. et al., 2023). A recent study on acetaminophen toxicity suggests that HMGB1 undergoes oxidative modification and can be used as pathological specific biomarkers and drug targets (Pirnie et al., 2023). A clinical experiment showed that with the increase of HMGB1 and age, the adverse functional outcome rate of ICH patients increased significantly, and the quantification of HMGB1 provided more accurate prognosis information after ICH (Lei et al., 2020). Hemmer et al. reported on a prospective, single-blind observational study designed to investigate the role of HMGB1 in aSAH. The study found that serum HMGB1 can serve as an independent biomarker for predicting delayed cerebral ischemia and elucidated its potential role in the sequelae of aSAH (Hemmer et al., 2022).

### 4.7 Ischemia-modified albumin (IMA)

IMA is more sensitive to ischemia and hypoxia in tissues and organs of the body, and we can generally detect it a few minutes after ischemia (Mangoni and Zinellu, 2024). Generally, IMA was initially recognized as a useful biomarker in context of ischemic diseases (Jena et al., 2017). Several studies have confirmed that free radicals increase in patients with cerebrovascular disease during episodes of local brain tissue ischemia, hypoxia, or local vascular reperfusion injury (Elshony et al., 2021). In the context of acute cerebrovascular disease, oxidative stress reactions occur progressively. The production of IMA can be partially attributed to the concurrent generation of reactive oxygen species and the disruption of the blood-brain barrier (Aycan et al., 2024). In a study, GAD et al. Confirmed the expression significance of IMA in ICH through clinical experiments, and also confirmed that it can be used to distinguish ICH from is (Gad et al., 2019). Mangoni et al. reported that in 17 previous clinical studies, the ima concentration gradually increased in patients with SAH, ICH and acute ischemic stroke through meta-analysis. In the sensitivity analysis, when individual studies were deleted in turn, the merged SMD did not change. It is suggested that IMA concentration may help to diagnose stroke and distinguish acute ischemic stroke, ICH and SAH (Mangoni and Zinellu, 2022).

### 4.8 Calcium binding protein S100

S100 protein is a kind of neural tissue protein. At present, nearly 20 kinds of S100 proteins have been found in different tissues, which can regulate intracellular and extracellular calcium ions with similar structure and function (Gayger-Dias et al., 2023). S100B is specifically found in astrocytes and glial cells within the central nervous system (Hernández-Ortega et al., 2024). Its content in brain tissue is much higher than that in other tissues, so it is considered to be brain specific protein (Santing et al., 2024). Generally, after brain injury, the expression of S100B increases with the occurrence of oxidative damage and neuroinflammation (Chong, 2016). Kellermann et al. reported that in patients with traumatic brain injury (TBI) and SAH, the concentrations of S100B in cerebrospinal fluid and serum were significantly higher in those with poor prognoses compared to those with favorable outcomes. Additionally, an initial serum S100B level greater than 0.7 μg/dl was associated with a 100% mortality rate, suggesting its potential utility in guiding treatment strategies for severe trauma (Kellermann et al., 2016).

### 4.9 Advanced oxidized protein (AOPP)

The principal component of AOPP is albumin that has been oxidized by free radicals. This compound can mediate lipid peroxidation, activate monocytes and NADPH oxidase, and promote the release of intracellular reactive oxygen species (de Brum and Bochi, 2024). It is one of the specific markers of protein oxidation (Lievykh et al., 2023). Rendevski et al. reported that AOPP may serve as a significant prognostic biomarker for ICH through modeling analyses (Rendevski et al., 2023). In another study, the expression of AOPPs in cerebrospinal fluid of 50 patients with aSAH at different time after hemorrhage was measured. The level of CSF AOPP at each time point after hemorrhage in patients with aSAH was independently correlated with the poor prognosis of 90 days follow-up, suggesting that AOPPs can be used as a potential biomarker for evaluating the prognosis of aSAH (Shen et al., 2022).

## 5 Haem peroxidase-cyclooxygenase superfamily

### 5.1 Myeloperoxidase (MPO)

MPO mainly exists in aniline blue granules of myeloid cells and is a specific indicator reflecting the activation of neutrophils and macrophages (Quinn et al., 2024). MPO especially reflects the function and activity of granulocytes (Tran et al., 2024). MPO itself is not inherently oxidative; however, under specific conditions, it catalyzes the production of reactive species such as 3-chlorotyrosine, which can induce oxidative damage (Wu TJ. et al., 2024). Zuo et al. performed experimental investigations to explore the involvement of MPO in oxidative stress after ICH. They examined the effects of an MPO inhibitor on neurobehavior in a rodent model of ICH and reported that inhibiting MPO can alleviate secondary injury after cerebral hemorrhage (Zuo et al., 2022b). Witsch et al. Conducted clinical observation on 100 patients with SAH (Witsch et al., 2022), and pointed out the diagnostic potential of mpo-dna complex and its possibility as a potential therapeutic target of aSAH. More specifically, Zheng et al. (Zheng et al., 2018) reported that by measuring serum myeloperoxidase (MPO) concentrations in 128 patients with cerebral hemorrhage and 128 controls, they assessed dependent variables such as neurological function, mortality, and adverse outcomes at various time points. The results indicated a significant increase in serum MPO concentration among patients with ICH. The study concluded that MPO concentration in ICH patients was positively correlated with hematoma volume and the NIHSS score. Furthermore, serum MPO detection significantly enhanced the ability to differentiate neurological function and prognosis related to hematoma in ICH patients.

Although the role of oxidative damage as a secondary injury in the pathophysiology of ICH is well established, the use of oxidative biomarkers for ICH diagnosis remains challenging due to the widespread occurrence of oxidative damage in various other diseases. Nevertheless, the correlation of oxidative biomarkers with the monitoring of ICH severity and prognosis is still acknowledged.

Although the role of oxidative stress as a mechanism of secondary injury in the pathophysiology of ICH is well established, the use of oxidative stress biomarkers for diagnosing ICH remains a significant challenge. This is because oxidative stress also occurs in various other diseases associated with energy metabolism dysfunction, neuroinflammation, tissue damage, and cell loss (Chen X. et al., 2024; Santos et al., 2024; Park et al., 2024). Additionally, several biomarkers mentioned above, which are metabolites or end products, may exhibit fluctuations in their levels due to the impact of other diseases on liver or kidney metabolic functions, further limiting the accuracy of these biomarkers. Therefore, improvements in methodology and the reliability of biomarkers are essential prerequisites for ensuring the reliability of biomarker detection. Moreover, a comprehensive analysis of various oxidative stress biomarkers following ICH may provide a more accurate reflection of the collective oxidative stress level. Currently, oxidative stress biomarkers are increasingly recognized as important tools for monitoring the severity and prognosis of ICH (Hu et al., 2016).

## 6 Assessment of antioxidant defense modifications as potential indirect markers of oxidative stress

Because reactive oxygen species are extremely unstable, they will react with cellular components or be catabolized rapidly, so it is difficult for existing clinical test methods to truly evaluate the content of ROS (Hosoki et al., 2023). Therefore, oxidative homeostasis is more indirectly assessed by evaluating oxidative stress products. To gain insights into the mechanisms of these changes, it is essential to examine the actual participants involved in the regulation of oxidants and antioxidants (Jomova et al., 2024). Some studies have attempted to utilize antioxidant defense to assess the extent of oxidative damage, which is considered a feasible oxidative biomarker (García-Giménez et al., 2024). The antioxidant system serves as an effective protective mechanism against potentially harmful oxidative damage. Antioxidant systems are primarily categorized into two types: enzymatic antioxidant systems, which include SOD, glutathione peroxidase, and catalase (CAT), and non-enzymatic antioxidant systems. The latter is further divided into water-soluble antioxidants, such as vitamin C (VC), and fat-soluble antioxidants, including carotene and vitamin E (VE) (Houldsworth, 2024).

### 6.1 Antioxidant enzymes

SOD is a key antioxidant enzyme in the body. It can cooperate with CAT and other enzymes to transform and decompose harmful superoxide free radicals into water that is harmless to the body, effectively reduce superoxide free radicals, and repair damaged cells and tissues in time, so as to improve the oxidative stress response (Militello et al., 2024). CAT is broadly present in animals, plants, and microorganisms (Afzal et al., 2023). Glutathione peroxidase (GPX) is a selenium containing antioxidant enzyme (Ikeda and Fujii, 2023). SOD, CAT, and GPX have distinct roles in both physiological and pathological processes. SOD degrades superoxide ions by converting them into ROS, while GPX and CAT are involved in the neutralization of these ROS, facilitating their conversion into oxygen and water (Grzeszczak et al., 2023).

A clinical study on ICH evaluated the oxidative stress marker SOD in cerebrospinal fluid and recorded the admission status, treatment outcomes, and degree of neurological impairment in ICH patients. The study found that SOD levels in the cerebrospinal fluid increased 24 h after the onset of ICH and were associated with poorer neurological outcomes (Krenzlin et al., 2020). Another clinical study involving 200 participants further validated the differences in serum levels of SOD and CAT between patients with ICH and healthy controls (Akyol et al., 2024). The study showed that GPX was measured by spectrophotometry and high performance liquid chromatography, respectively. It was found that SAH was accompanied by the change of antioxidant capacity in plasma, including the increase of GPX activity in the first day, and then its decrease, suggesting that the level of plasma GPX can support the monitoring of patients’ clinical status (Jarocka-Karpowicz et al., 2020b). Zhang et al. reported on a clinical study involving 116 patients with severe ICH admitted to the ICU from June 2018 to June 2020. The study observed significantly elevated levels of SOD and GPX in the plasma of ICH patients, indicating the occurrence of oxidative damage *in vivo* following ICH (Zhang J. et al., 2021).

### 6.2 Non-enzymatic antioxidants

Compared to enzymatic antioxidants that are directly involved in the metabolism of ROS, non-enzymatic antioxidants primarily function as ROS scavengers (Rudenko et al., 2023). These antioxidants terminate the redox reaction by eliminating the intermediates of free radicals, and also generate new free radicals (Dyachenko and Bel’skaya, 2023). Vitamin E can be converted into tocopherol carboxyl radical, which has relatively low activity. It also needs to be antioxidant and scavenged by another kind, such as ascorbic acid (vitamin C) (Feng et al., 2023). If the clearance of tocopherol carboxyl radical is delayed, it will promote lipid peroxidation (Lian et al., 2023). Vitamin C is an important antioxidant because ascorbic acid free radicals are usually disproportionately converted to ascorbic acid and dehydroascorbic acid (Fiorentino et al., 2024). In recent years, the antioxidant effect of natural antioxidants has become a research hotspot. The study measured the plasma levels of vitamins A, E, and C in patients with ICH using spectrophotometry. Compared to healthy controls, patients with ICH exhibited elevated levels of these vitamins, suggesting an association with oxidative stress injury and potentially reflecting the prognostic outcomes for individuals affected by ICH (Jarocka-Karpowicz et al., 2020b).

### 6.3 Total antioxidant capacity (T-AOC)

T-AOC is a comprehensive index to measure the functional status of antioxidant system, and its content can reflect the compensatory ability of antioxidant system against external stimuli and the status of free radical metabolism (Hu W. et al., 2023). T-AOC is defined as the total antioxidant capacity of all antioxidants in the sample to be tested. It is a clinical biochemical examination index, which is measured by detecting the total amount and activity of antioxidants in blood, urine or other body fluids (Mei et al., 2021). Animal research models show that ICH is usually accompanied by the increase of total antioxidant capacity. It is observed that the T-AOC in the serum of ICH group increases at 24 and 72 h, which may be the physiological mechanism against the increase of ROS production (Mazhar et al., 2023). In a study by Zhang et al., it was reported that the T-AOC and other indicators of oxidative damage in the experimental group were elevated above the average values. This finding suggests an enhancement in oxidative stress processes within this group (Zhang J. et al., 2021). However, an increase in serum antioxidant capacity may not represent optimal state, as it could indicate the presence of ongoing pathological processes in the body. Additionally, a subsequent decrease in these levels may reflect a response to the reduction of active substances (Morimoto et al., 2019). Consequently, new insights have emerged regarding the survival strategies for patients with ICH and the management of oxidative stress: specifically, the importance of restoring oxidative balance while avoiding excessive activation of the immune system.

### 6.4 Total antioxidant status (TAS)

TAS is an indicator of the reserve state of reduced complex, which can more accurately assess the body’s oxidative stress (Oktay et al., 2024). The general significance of TAS assessment is to better understand the ability to resist ROS attack on cells (Ghasemi et al., 2023). In a few published studies using TAS to measure the antioxidant potential of diseases, animal models showed that the occurrence of SAH was often accompanied by the increase of TAS, suggesting that this may be a response to the increase of ROS (Senol et al., 2021). Masomi et al. conducted a clinical study involving 48 patients, which revealed that the TAS of cerebrospinal fluid increased further by the seventh day following ICH. The plasma TAS levels were found to be independently associated with poor prognostic outcomes in patients with ICH (Masomi-Bornwasser et al., 2021b).

## 7 Future treatment directions

Currently, the primary treatment options available in clinical practice include hematoma evacuation and edema management. While numerous clinical trials have been conducted to assess these treatments, there is no conclusive evidence suggesting that surgical interventions can improve neurological deficits following ICH (Hanley et al., 2019). Vascular edema induced by ICH has also attracted the attention of researchers. For instance, there have been efforts to investigate the use of glibenclamide for edema treatment, with some progress observed in clinical trials (Zhao et al., 2022). However, it is interesting to note that preclinical studies on the efficacy of glibenclamide in treating ICH seem to present conflicting results (Kung et al., 2021). Additionally, emerging therapeutic strategies such as miRNA-based treatments and exosome therapies have gained attention. The use of miRNA mimics or antagonists to improve outcomes after experimental brain hemorrhage has been validated in numerous studies (Wu X. et al., 2024; Hou et al., 2024). This suggests that, with proper delivery methods, miRNA-based therapy may hold potential for improving clinical outcomes in ICH. Exosome therapy, a more recent approach, has been shown in preclinical studies to mitigate inflammatory damage following ICH, thereby offering neuroprotective effects, and its potential has been substantiated (Jiang et al., 2024; Nan et al., 2025). However, both miRNA and exosome therapies have yet to be evaluated in clinical trials. In summary, while hematoma evacuation and edema management have uncertain clinical efficacy, emerging therapies remain in the early stages of research and face challenges such as high costs, indicating that treatment strategies for ICH still require further optimization.

Numerous studies have demonstrated that oxidative damage is a critical pathological mechanism involved in ICH injury. Consequently, oxidative stress has emerged as a significant area of research in the treatment and prevention of ICH.

### 7.1 Targeted oxidative stress may be a potential treatment for ICH

The existing treatment strategies for ROS to treat ICH can be divided into “upstream” (reducing reactive oxygen species) or “downstream” (reactive oxygen species neutralization) strategies. Although a large number of studies in animal and cell models have shown the positive effect of antioxidants on ICH (Tang et al., 2023c; Zhang Z. et al., 2024; Liu et al., 2024; Endo et al., 2024), the same sufficient data support has not been obtained in clinical trials, which may be due to the unclear understanding of the complex mechanism of secondary injury after ICH. Recent studies on practical drugs for targeted treatment of oxidative stress in ICH treatment mainly include rosiglitazone, edaravone, atorvastatin, minocycline and deferoxamine (Luo et al., 2021; Wang Z. et al., 2024; Sun et al., 2023; Gan et al., 2023; Daou et al., 2019).

Rosiglitazone, by activating PPARγ, reduces the expression of NF-κB and MMP9, thereby alleviating oxidative stress damage following ICH. However, this research is currently limited to animal studies, and no clinical trial results are available. Edaravone, a free radical scavenger, has demonstrated certain antioxidant effects. Animal experiments have shown that edaravone can attenuate oxidative damage to brain tissue in mice following ICH through the OS/MMP9/β-DG pathway (Shan et al., 2021). On the other hand, significant progress has been made in clinical trials involving edaravone. In a clinical trial, Shan et al. reported that edaravone inhibits lipid peroxidation during the process of free radical scavenging, leading to anti-inflammatory protection of neural cells, reducing neuronal damage, and improving neurological function and prognosis after ICH (Munakata et al., 2009). Deferoxamine (DFX) works by inhibiting redox reactions through competitive binding with iron ions, thereby reducing free radical production following ICH (Wan et al., 2025). A phase II clinical trial involving 324 patients with spontaneous ICH demonstrated that high-dose deferoxamine effectively reduces oxidative stress-induced damage and improves clinical outcomes (Yeatts et al., 2013). Minocycline, an MMP inhibitor, has notable antioxidant properties, lowering ROS levels in brain tissue after ICH, possibly through the clearance of free radicals (Zheng et al., 2022a). Some studies suggest that minocycline has significant neuroprotective effects in acute stroke and may be a promising therapeutic agent (Malhotra et al., 2018). Previous animal studies have found that atorvastatin protects neurons in ICH rats by reducing MMP-9-induced brain injury (Cui et al., 2012). Interestingly, however, the effectiveness of this protective effect in clinical trials has not yet been confirmed (Lei et al., 2014) (Table 2).

**TABLE 2:** The development for antioxidative treatment of ICH.

Drug name	Different types of intracerebral hemorrhage	Action mechanism	Research progress	References
Rosiglitazone	ICH	Activation of PPARγ reduces the expression of NF-κB and MMP9	Animal test phase	Luo et al. (2021)
Edaravone	ICH	Free radical scavenger	Preliminary clinical trial	Munakata et al. (2009)
Deferoxamine mesylate	ICH	Iron-chelator	Phase II clinical trial	Yeatts et al. (2013)
Minocycline	ICH	Free radical scavenger	Preliminary clinical trial	Malhotra et al. (2018)
Atorvastatin	ICH	Inhibition of MMP-9 expression	Animal test phase	Lei et al. (2014)

A possible explanation for the poor effectiveness of ROS targeted therapy in ICH has been proposed, because it is easier to inhibit the production of ROS than to degrade and neutralize ROS, and oxidative damage and inflammatory damage are often accompanied in the secondary injury of ICH. Upstream strategies targeting the inhibition of MPO, COX-2, and MMP-9 have been proposed. Hua et al. reported that Y-2, a drug currently undergoing Phase III clinical trials for ischemic stroke in China, may also exert therapeutic effects in ICH. Specifically, Y-2 appears to reduce the levels of MPO, pro-inflammatory mediators, and oxidative products in the brain tissue surrounding the hematoma core (Hua et al., 2021). Tang et al. investigated the pathological mechanisms underlying ICH and highlighted the critical role of Nrf2 within the Nrf2/ARE signaling pathway (Yan et al., 2023c). This pathway has been shown to attenuate the levels of serum superoxide dismutase (SOD) and glutathione peroxidase (GSH-PX) following ICH, suggesting that targeting Nrf2 may represent a promising strategy to mitigate oxidative stress. In addition to the Nrf2 pathway, recent studies have highlighted the significant role of the Wnt signaling pathway in oxidative stress following ICH. Low-density lipoprotein receptor-related protein 6 (LRP6), a transmembrane cell surface protein that induces the canonical Wnt signaling pathway, plays a significant role in the development and metabolism of the nervous system. It is involved in regulating neuronal processes such as differentiation and synaptic plasticity, and is particularly important in the context of oxidative damage (Clark-Corrigall et al., 2023; Wang Y. et al., 2020). Jin et al. evaluated the pathological processes of oxidative damage after ICH and reported the critical involvement of the Wnt signaling pathway in this process (Jin et al., 2022). Furthermore, intervention targeting LRP6 alleviated oxidative damage following ICH, suggesting that modulating LRP6 and the Wnt pathway may be an effective strategy for mitigating oxidative stress. Additionally, Riitano et al. reported the effects of methyl-β-cyclodextrin on the Wnt/β-catenin signaling pathway, demonstrating that it inhibited the phosphorylation of β-catenin (Riitano et al., 2020). This finding suggests that methyl-β-cyclodextrin, in combination with statins, could represent a potential therapeutic strategy for intervention. Although the effects of this drug have not been explored in the context of oxidative damage, it provides a potential avenue for further investigation. Recent studies have also confirmed the role of the Notch signaling pathway (Notch1, Notch4) in the development of the vascular system, as well as in neurovascular formation and antioxidative effects (Jin et al., 2011; Muley et al., 2022). Although the role of Notch signaling in oxidative stress after ICH has not been extensively studied, targeting the Notch pathway may offer new insights for future research into therapies for oxidative stress following ICH.

### 7.2 Potential of antioxidants to prevent ICH

Antioxidants have been widely recognized and used to improve the body’s immunity (Halliwell, 2024; Yang et al., 2024). In addition, the application of antioxidant therapy in non communicable diseases has also achieved exact curative effect (Gao et al., 2023; Lu X. et al., 2023; Feng et al., 2024b; Hu Y. et al., 2023). In animal model experiments, antioxidants are often chemically synthesized. Because of their potential harmful effects, they are rarely used in clinical practice. Therefore, antioxidants from dietary sources may be a key factor in the prevention of ICH. Food sources of oxidants usually include fruits, vegetables and some herbs. Zheng et al. identified several medicinal plants with potential therapeutic benefits, categorizing them into groups such as polyphenols and phenolic compounds, terpenoids, and alkaloids. Examples include garlic, Ligusticum chuanxiong, Cordyceps sinensis, and bitter gourd. These plants have been reported to exert significant effects in animal models of ICH, primarily through their antioxidant properties (Zheng et al., 2022b). Several studies have demonstrated that cordycepin improves brain hemorrhage-induced neurological and cognitive impairments in mouse models by reducing oxidative stress (An et al., 2022). Allicin has been shown to alleviate oxidative stress after ICH by inhibiting MDA expression and increasing SOD levels (Atef et al., 2023). Gastrodin, a phenolic glycoside derived from the plant Gastrodia elata, has been reported by Liu et al. to reduce oxidative damage caused by ICH by suppressing levels of ROS, 8-OHDG, 3-nitrotyrosine, and MDA, while simultaneously increasing the activities of GSH-Px and SOD (Iu et al., 2020). Puerarin, an extract from the plant Pueraria lobata, has been shown to reduce oxidative damage caused by ICH by lowering the activity of 8-OHdG and ROS (Zeng et al., 2021). Naringin, a flavonoid found in fruits such as grapefruit, has been found to effectively reverse oxidative damage and elevate endogenous antioxidant levels when administered to ICH rats (Singh et al., 2017). Artemisinin, extracted from the plant Artemisia annua, not only combats malaria but also exerts a neuroprotective effect by upregulating the expression of the neural cell adhesion molecule L1, contributing to its antioxidant properties and brain protection (Wang J. et al., 2022). Similarly, baicalin, derived from the herb Scutellaria baicalensis, has been shown to reduce oxidative stress levels in ICH rats, with its mechanism involving the miR-106a-5p/PHLPP2 axis to activate the Nrf2/ARE pathway (Tang et al., 2023d). Ursolic acid, found in plants like hawthorn and gardenia, has been demonstrated in animal studies to effectively enhance the activity of GSH, CAT, and SOD, thereby mitigating oxidative stress following SAH (Zhang et al., 2014). Tetramethylpyrazine nitrate, extracted from Chuanxiong (Ligusticum chuanxiong), possesses antioxidant properties. Wu et al. reported that it can alleviate oxidative damage after SAH, likely through the modulation of the Nrf2/HO-1 pathway (Wu et al., 2019). Isorhynchophylline (IRN), a compound found in the herb Uncaria rhynchophylla, has been shown to exert neuroprotective effects in ICH rats by modulating oxidative stress levels (Zhao et al., 2021). Polydatin, primarily extracted from grapes, red wine, and Crocus sativus, has been found in a recent study to exert antioxidant effects in SAH rats by downregulating NO and MDA while upregulating SOD, GSSG, and GSH (Zhao et al., 2020). Phillyrin, a natural extract, increases Nrf2, HO-1, and SOD-1 levels in the brain tissue of ICH mice, likely through the modulation of the Nrf2 signaling pathway (Guo et al., 2021). Epigallocatechin gallate (EGCG), a major catechin in green tea, has been confirmed to suppress oxidative stress in ICH rats by activating the Keap1/P62/Nrf2 pathway (Hao et al., 2024). Sosa et al. reported similar antioxidant effects for black tea (Sosa et al., 2018). Duan et al. found that Momordica charantia polysaccharide (MCP) can scavenge ROS in the brain tissue of ICH rats and reduce neuronal death, possibly through the inhibition of the JNK3 signaling pathway (Duan et al., 2015) (Table 3).

**TABLE 3:** Antioxidant activity of nature product in ICH related studies.

Food sources	Chemical composition	Different types of intracerebral hemorrhage	Action mechanism	References
Cordyceps sinensis	Cordycepin	ICH	Increase endogenous antioxidant levels	An et al. (2022)
Garlic	Allicin	ICH	Inhibiting MDA expression and increasing SOD levels	Atef et al. (2023)
Rhizoma Gastrodiae	Gastrodin	ICH	Increase GSH-Px and SOD activity	Iu et al. (2020)
Pueraria lobata	Puerarin	ICH	Reduce the activity of 8-OHdG and ROS	Zeng et al. (2021)
Grapes, cherries, tomatoes, beans	Naringin	ICH	Enhance endogenous antioxidant levels	Singh et al. (2017)
Artemisia annua	Artemisinin		Upregulation of the L1CAM	Wang et al. (2022d)
Scutellaria baicalensis	Baicalein	ICH	Mir-106a-5p/phlpp2 axis activates Nrf2/are pathway	Tang et al. (2023d)
Hawthorn, cranberry, gardenia	Ursolic Acid	SAH	Increase GSH,CAT and SOD activity	Zhang et al. (2014)
Chuanxiong	Tetramethylpyrazine nitrone	SAH	Upregulation of the Nrf2/HO-1 pathway	Wu et al. (2019)
Uncaria rhynchophylla	IRN	ICH	Reduction in the production of ROS, 4-HNE, and MDA	Zhao et al. (2021)
Grapes, red wine, Crocus sativus	Polydatin	ICH	Downregulation of NO and MDA, and upregulation of SOD, GSSG, and GSH	Zhao et al. (2020)
Forsythia suspensa	Phillyrin	ICH	Activation of the Nrf2 signaling pathway	Guo et al. (2021)
Green tea, black tea	EGCG	ICH	Activation of the Nrf2 signaling pathway	Hao et al. (2024), Sosa et al. (2018)
Balsam pear	Momordica charantia polysaccharide	ICH	Inhibition of the JNK3 signaling pathway	Duan et al. (2015)

While these natural products demonstrate promising antioxidant activity in experimental studies, further research is needed to validate their properties. In the next phase, integrating genomics and proteomics approaches could help elucidate the specific molecular targets of these natural products and explore their potential for future therapeutic applications.

The antioxidant potential of many foods is also shown under pathological conditions, such as nuts, goat milk, blueberries, etc. (Lorenzon Dos Santos et al., 2020; Feng and Wang, 2020; Martini et al., 2023). However, the added ingredients of foods tend to be cytotoxic, and most evidence points out that oxidative stress is the main mechanism of toxicity (Medina-Reyes et al., 2020; Martínez Leo et al., 2021). Therefore, the intake of naturally grown products without chemicals, which show additional antioxidant properties, may be a potential prevention method.

## 8 Conclusion

The primary damage resulting from ICH is largely attributable to secondary injury processes, which include local tissue damage and programmed cell death. Experimental evidence strongly supports the involvement of oxidative stress, stemming from an imbalance in redox homeostasis, in the pathophysiology of ICH. Consequently, the assessment of oxidative stress biomarkers holds promise as a means to elucidate the underlying mechanisms of ICH, offer diagnostic and prognostic insights, and guide the identification of potential therapeutic targets for antioxidant-based interventions. However, despite the recognized potential of oxidative damage in ICH prediction and treatment, clinical studies have shown limited reproducibility in oxidative stress marker measurements. This inconsistency is largely due to variability in disease subtypes, as well as limitations in sample collection, storage, and pretreatment protocols. Furthermore, the complex interplay of other mechanisms contributing to ICH-induced injury remains incompletely understood, leading to conflicting findings and inconclusive results. While comprehensive analysis of various oxidative stress biomarkers after ICH may provide an indication of oxidative damage levels, significant improvements in methodology are required to enhance the reliability and accuracy of these measures.
